# Correlation Between Fat Attenuation Index and Major Adverse Cardiovascular Events: A Systematic Review and Meta-Analysis

**DOI:** 10.31083/RCM46683

**Published:** 2026-04-24

**Authors:** Yingzi Tan, Yaojian Wang, Boya Zhao, Yuerong Jiang, Keji Chen

**Affiliations:** ^1^Graduate School, Beijing University of Chinese Medicine, 100029 Beijing, China; ^2^National Clinical Research Center for Chinese Medicine Cardiology/State Key Laboratory of Traditional Chinese Medicine Syndrome, Xiyuan Hospital, China Academy of Chinese Medical Sciences, 100091 Beijing, China

**Keywords:** adipose tissue, cardiovascular diseases, systematic review, meta-analysis

## Abstract

**Background:**

Numerous previous studies have examined the relationship between the fat attenuation index (FAI) and major adverse cardiovascular events (MACE), reporting inconsistent findings.

**Methods:**

We conducted a systematic search of four databases (PubMed, Embase, Web of Science, and the Cochrane Library) for cohort, case–control, and cross-sectional studies evaluating the association between FAI and MACE incidence. The outcomes were defined as the correlations between MACE and FAI, including total FAI, FAI of the right coronary artery (RCA), FAI of the left circumflex coronary artery (LCX), and FAI of the left anterior descending (LAD) artery. FAI was analyzed both as a continuous and categorical indicator. Two researchers determined the final inclusion of the literature based on the inclusion and exclusion criteria and completed the data extraction. Study quality was assessed using the Newcastle–Ottawa Scale (NOS). RevMan 5.4 was used to conduct heterogeneity tests, perform statistical pooling, and generate forest plots. Hazard ratios (HRs) were used to estimate the association between FAI and MACE risk. STATA16.0 (StataCorp LLC, College Station, TX, USA) was used to generate funnel plots, and the Egger test was applied to evaluate publication bias.

**Results:**

A total of 22 studies involving 10,224 participants were included: 17 cohort studies, 1 cross-sectional study, and 4 case–control studies. The meta-analysis results suggested that there was a significant correlation between MACE and total FAI (FAI as a categorical variable: HR = 2.77, 95% confidence interval (CI) = 2.22–3.46; *p* < 0.00001; FAI as a continuous variable: HR = 1.15, 95% CI = 1.05–1.26; *p* = 0.003). There was also a significant association between MACE risk and FAI for the RCA (FAI as a categorical variable: HR = 2.10, 95% CI = 1.58–2.79; *p* < 0.00001; FAI as a continuous variable: HR = 1.06, 95% CI = 1.04–1.08; *p* < 0.00001), a significant correlation between the risk of MACE and FAI for the LAD (FAI as a categorical variable: HR = 2.76, 95% CI = 1.93–3.97; *p* < 0.00001; FAI as a continuous variable: HR = 1.09, 95% CI = 1.06–1.11; *p* < 0.00001), a significant correlation between the risk of MACE and FAI for the LCX branch (FAI as a categorical variable: HR = 2.68, 95% CI = 1.24–5.80; *p* = 0.01; FAI as a continuous variable: HR = 1.07, 95% CI = 1.05–1.10; *p* < 0.00001). Meanwhile, individuals with elevated FAI levels had a significantly increased risk of developing MACE.

**Conclusion:**

The results of this meta-analysis show a significant association between FAI and MACE. Higher FAI values are associated with significantly higher risks of MACE. These results suggest that FAI may serve as an imaging indicator for predicting the risk of MACE.

**PROSPERO Registration:**

CRD420250652674, https://www.crd.york.ac.uk/PROSPERO/view/CRD420250652674.

## 1. Introduction

Cardiovascular diseases (CVDs) rank as the top global cause of mortality and pose a significant hazard to human health [1, 2]. In the field of CVD prevention and treatment, accurate prediction of the risk of adverse events and guidance for early prevention in patients have always been important goals. Recent technological breakthroughs in imaging have significantly facilitated the diagnostic process and clinical management of CVDs. Currently, CT angiography (CCTA) is widely endorsed as a first-line research tool for identifying Coronary Artery Disease (CAD). The fat attenuation index (FAI) obtained from coronary CCTA has received increasing attention as an emerging, practical, and non-invasive biomarker to reflect coronary artery inflammation in CVD risk assessment [3]. It provides a quantitative basis for the precise diagnosis and treatment of CVD by capturing the functional and metabolic abnormalities of perivascular adipose tissue (PVAT) [4]. In clinical practice, FAI can identify the potential risk of CVD in asymptomatic high-risk populations [5]. Clinical studies have preliminarily confirmed that CVD patients with high FAI values have a significantly higher risk of developing major adverse cardiovascular events (MACE) compared to patients with low FAI values [6]. In addition, FAI can also be used to evaluate the plaque reversal effect of drug therapy (such as statins) or interventional therapy in CVD patients, providing dynamic monitoring indicators for personalized adjustment of treatment plans [7].

Although a substantial number of studies have focused on the relationship between FAI and cardiovascular adverse events, the conclusions of these studies are not entirely consistent. Some studies suggest a significant positive link between FAI and cardiovascular adverse events, with elevated FAI indicating a higher risk of cardiovascular adverse events [8, 9], while others suggest the opposite [10]. The inconsistency of these results not only confuses clinicians when using FAI for cardiovascular risk assessment, but also affects its use in clinical practice. A meta-analysis is a statistical method that quantitatively synthesizes the results of different studies focusing on the same scientific question. It can obtain larger and higher-quality clinical research evidence by merging and analyzing data from multiple small-sample randomized controlled trials (RCTs). In this study, we sought to systematically evaluate the relationship between FAI and MACE through meta-analysis to clarify its potential value in predicting MACE. By comprehensively analyzing data from multiple related studies, we hope to provide a more accurate risk assessment indicator for clinical practice and a scientific basis for future research investigations.

## 2. Methods

This meta-analysis was strictly performed in accordance with the PRISMA (Preferred Reporting Items for Systematic Reviews and Meta-Analyses) guidelines [11]. The complete PRISMA checklist is provided in **Supplementary Material 1**. This research protocol has been registered in PROSPERO (registration number: CRD42022346488, https://www.crd.york.ac.uk/PROSPERO/recorddashboard)

### 2.1 Inclusion and Exclusion Criteria

Two investigators (YT and YJ) independently screened the literature based on rigorous inclusion and exclusion criteria. We developed the inclusion criteria as follows: (1) Population types: Patients diagnosed with or without CVD, regardless of age, sex, disease course, region, nationality, and race; (2) Types of outcomes: Correlation between outcome indicators FAI and MACE. MACE includes: a. cardiovascular death; b. Non-fatal myocardial infarction; c. Non-lethal stroke; d. Re-hospitalization for heart failure; e. Severe arrhythmia; f. Recurrent angina pectoris; g. Myocardial Revascularization. (3) Types of study design: Prospective or retrospective observational study. (4) Exposure factor: FAI score. Exclusion criteria were as follows: (1) Studies in which a hazard ratio (HR) could not be calculated; (2) Duplicate articles or failure to retrieve the full-text literature; (3) Literature that could not be extracted from the raw data.

### 2.2 Search Strategy

Cohort studies, case-control studies, and cross-sectional studies on the relationship between FAI and the incidence of MACE were searched in the relevant databases, including PubMed, Embase, Web of Science, and the Cochrane Library. Retrieval was conducted from the inception of the databases to August 25, 2025. The search strategy was developed based on the Cochrane Handbook. Search terms are as follows: “fat attenuation index”, “adverse cardiac events”, “myocardial infarction”, “stroke”, “heart failure”, “death, sudden, cardiac”, “MACE”, “myocardial revascularization”, “severe arrhythmia”, “recurrent angina pectoris”. We used a combination of MeSH terms and free-text terms to conduct the search. MeSH words have clear conceptual directionality and a synonymous extension function, which can avoid systematic omissions caused by differences in synonyms and synonyms within the discipline. Free text words cover commonly used abbreviations, non-standardized expressions, and colloquial names that may appear in research works, which can supplement literature not covered by MeSH words. The combination of the two can achieve a complementary effect of “standardized retrieval + flexible supplementation”, ensuring the systematic and accurate retrieval. We also search for references in relevant papers to avoid omissions. The detailed search strategies for each database are shown in **Supplementary Material 2**.

### 2.3 Literature Screening and Data Extraction

Two researchers (YT and YJ) independently screened the literature based on the inclusion and exclusion criteria. If there were differences in opinions during the screening process, they were resolved through discussion. If opinions could not be unified, a third party (KC) made the judgment. Records from databases were managed and screened using NoteExpress. The data extraction items were as follows: (1) basic information of literature, such as the first author’s name and year of publication; (2) characteristics of the research object, such as sample size, types of study design, gender, and age. The two researchers (YT and YJ) worked independently to extract the following data from the included studies. The discrepancies were settled via discussion.

### 2.4 Risk of Bias Assessment 

According to the Newcastle-Ottawa Scale (NOS), two researchers (YT and YJ) separately appraised the risk of bias of the included studies. A NOS score >7 indicates high-quality research, 5–7 indicates moderate-quality research, and 0–4 indicates low-quality research. In the event of a dispute between the two researchers, the third party (KC) mediated.

### 2.5 Data Analysis and Synthesis

We used RevMan 5.4 software (The Cochrane Collaboration, Copenhagen, Capital Region, Denmark) to analyze and synthesize data. The HR was adopted as the effect measure, with its 95% confidence interval (CI). If only the beta value was given in the original study, it was converted to the HR value for analysis, and the conversion formula was HR = exp (beta). For the conversion of 95% CIs of HR, we first extracted the SE of beta from each study; if SE(beta) was not directly provided, it was imputed using the reported 95% CI or *p* value of beta based on the formula SE(beta) = (upper 95% CI – lower 95% CI) / (2 × 1.96) or SE(beta) = |beta| / Z (where Z is the Z-score corresponding to the reported *p* value). In statistical models, when FAI is used as a continuous variable, the HR represents the proportion of increased risk of MACE for each increase in Hounsfield unit (HU). In statistical models, when FAI is used as a categorical variable, the HR represents the risk ratio of MACE for a given category (such as the high FAI group) relative to the reference category (such as the low FAI group). We assessed group heterogeneity using the I^2^ test and Cochran’s Q test. The fixed-effects model was adopted for analysis when *p* > 0.1 and I^2^ ≤ 50%, suggesting modest heterogeneity. If *p* < 0.1 and I^2^ > 50%, the heterogeneity is considered to be significant, and sensitivity analysis was employed to address the issue. After excluding the influence of significant clinical heterogeneity, the random-effects model was selected for the trial. STATA16.0 (StataCorp LLC, College Station, TX, USA) was employed to draw funnel plots for indicators with more than 10 included studies, and the Egger test was used to assess publication bias. If there was publication bias, a trim-and-fill technique was used for correction.

## 3. Results

**Fig. 1.**
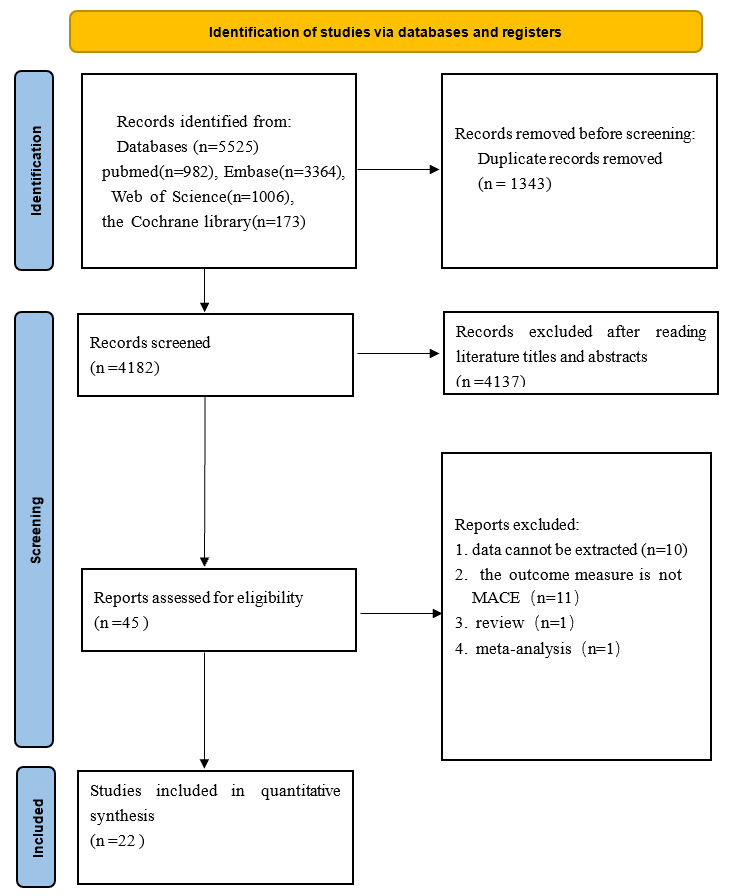
**The flow diagram**. MACE, major adverse cardiovascular events.

We retrieved 5525 studies in total from four databases: 982 in PubMed, 3364 in Embase, 1006 in Web of Science, and 173 in the Cochrane Library. After importing all texts into NoteExpress 4.2.0.10271(Beijing Aegean Technology Co., Ltd., Chaoyang, Beijing, China) and removing duplicate texts, 4182 texts were obtained. After reading the title and abstract, 45 studies were obtained by removing the obviously ineligible ones. Then, 45 research articles were read in detail after being downloaded using different methods. 23 papers were excluded: HR could not be calculated from 10 articles [8, 9, 12, 13, 14, 15, 16, 17, 18, 19], the outcome measure of 11 articles was not MACE [20, 21, 22, 23, 24, 25, 26, 27, 28, 29, 30], 1 article is a meta-analysis [31], and 1 article is a review [32]. Finally, 22 articles [10, 33, 34, 35, 36, 37, 38, 39, 40, 41, 42, 43, 44, 45, 46, 47, 48, 49, 50, 51, 52, 53] were screened. The process of document screening is presented in Fig. 1.

### 3.1 Main Characteristics of Included Studies

The 22 included articles [10, 33, 34, 35, 36, 37, 38, 39, 40, 41, 42, 43, 44, 45, 46, 47, 48, 49, 50, 51, 52, 53] were published from 2021 to 2025. 13 studies [36, 37, 38, 39, 40, 41, 42, 47, 49, 50, 51, 52, 53] are from China, 3 studies [43, 46, 48] from Japan, 2 studies [44, 45] from Italy, while the rest [10, 33, 34, 35] are from India, the UK, the USA, and the Netherlands. A total of 10,224 patients were included. The maximum sample size is 3393, and the minimum sample size is 50. 17 studies [10, 34, 35, 36, 38, 41, 42, 44, 45, 46, 47, 48, 49, 50, 51, 52, 53] used MACE as the endpoint, 2 studies [37, 40] used acute coronary syndrome (ACS) as the endpoint 1 study [43] used non-infarct-related territory unrecognized myocardial infarction (non-IR UMI) as the endpoint, 1 study [39] used unstable angina as the endpoint, 1 study [33] used acute coronary events as the endpoint, 1 study [46] used major adverse cardiac and cerebrovascular events (MACCE) as the endpoint. Basic information and characteristics of the included trials are presented in Table 1 (Ref. [10, 33, 34, 35, 36, 37, 38, 39, 40, 41, 42, 43, 44, 45, 46, 47, 48, 49, 50, 51, 52, 53]).

**Table 1.** **General characteristics of the included review**.

Study	Publication year	Country	Study type	Disease	Age	Cases (male/female)	Endpoint
Biradar, B *et al*. [33]	2025	India	Cohort study	CAD	59.23 ± 10.8	120 (71/49)	acute coronary events
Chan, K *et al*. [34]	2024	UK	Cohort study	CAD	—	3393 (1914/1479)	MACE
Chatterjee, D *et al*. [10]	2021	USA	Cohort study	known or suspected CAD	—	344 (—)	MACE
Coerkamp, C *et al*. [35]	2024	the Netherlands	Cohort study	suspected CAD	62 ± 7.7	50 (20/30)	MACE
Dai, X *et al*. [36]	2022	China	Cohort study	chest pain	64.8 ± 11.0	263 (186/77)	MACE
Huang, M *et al*. [37]	2023	China	Case control study	ACS	59.3 ± 12.3	70 (57/13)	ACS
Huang, S *et al*. [38]	2024	China	Cohort study	after aortic valve replacement	—	139 (87/52)	MACE
Li, D *et al*. (1) [39]	2025	China	Case control study	unstable angina	64 ± 11.9	130 (75/55)	unstable angina
Li, D *et al*. (2) [40]	2025	China	Cohort study	stable angina pectoris	—	278 (156/122)	ACS
Liu, M *et al*. [41]	2024	China	Cohort study	type 2 diabetes mellitus (T2DM)	61.79 ± 9.86	304 (157/147)	MACE
Luo, C *et al*. [42]	2024	China	Cohort study	CAD	—	790 (527/263)	MACE
Matsuda, K *et al*. [43]	2021	Japan	Case control study	ACS	—	158 (121/37)	non-IR UMI
Pergola, V *et al*. [44]	2022	Italy	Cohort study	no symptom or chest pain	55.5 ± 11.9	371 (237/134)	MACE
Sansonetti, A *et al*. [45]	2025	Italy	Cohort study	after heart transplant	55.5 ± 13	101 (63/38)	MACE
Sayama, K *et al*. [46]	2023	Japan	Case control study	takotsubo cardiomyopathy	71 ± 13	52 (10/42)	MACCE
Sun, X *et al*. [47]	2024	China	Cohort study	suspected CAD	69.14 ± 9.65	260 (153/107)	MACE
Teng, Y *et al*. [48]	2024	Japan	Cohort study	CCS	—	181 (145/36)	MACE
Xie, Y *et al*. [49]	2024	China	Cohort study	lung cancer	—	697 (345/352)	MACE
Xu, Q *et al*. [50]	2024	China	cross-sectional study	thoracic malignancies	—	1543 (638/807)	MACE
Yu, Y *et al*. (1) [51]	2024	China	Cohort study	suspected CAD	60.6 ± 9.9	260 (101/159)	MACE
Yu, Y *et al*. (2) [52]	2025	China	Cohort study	suspected CAD	40.18	503 (386/117)	MACE
Zhang, X *et al*. [53]	2024	China	Cohort study	chest pain	64.95 ± 10.82	217 (139/78)	MACE

ACS, acute coronary syndrome; CAD, Coronary Artery Disease; CCS, Chronic Coronary Syndromes; MACCE, major adverse cardiac and cerebrovascular events; non-IR UMI, non-infarct-related territory unrecognized myocardial infarction; MACE, major adverse cardiovascular events.

### 3.2 Methodological Quality Evaluation

In the risk of bias assessment, all included references received a score of 7 or higher, indicating that the quality of the literature was high. The results are shown in the following table (Table 2, Ref. [10, 33, 34, 35, 36, 38, 40, 41, 42, 44, 45, 47, 48, 49, 51, 52, 53]; Table 3, Ref. [37, 39, 43, 46]; Table 4, Ref. [50]).

**Table 2.** **Quality evaluation form for cohort research**.

Study	Year	Selectiveness	Comparability	Exposure	Total score
Biradar, B *et al*. [33]	2025	4	2	3	9
Chan, K *et al*. [34]	2024	4	2	3	9
Chatterjee, D *et al*. [10]	2021	4	0	3	7
Dai, X *et al*. [36]	2022	4	2	3	9
Huang, S *et al*. [38]	2024	4	1	3	8
Li, D *et al*. (2) [40]	2025	4	2	3	9
Liu, M *et al*. [41]	2024	4	2	3	9
Luo, C *et al*. [42]	2024	4	2	3	9
Pergola, V *et al*. [44]	2022	4	2	3	9
Sansonetti, A *et al*. [45]	2025	4	2	3	9
Sun, X *et al*. [47]	2024	4	2	3	9
Teng, Y *et al*. [48]	2024	4	2	3	9
Xie, Y *et al*. [49]	2024	4	2	3	9
Yu, Y *et al*. (1) [51]	2024	4	2	3	9
Yu, Y *et al*. (2) [52]	2025	4	2	3	9
Zhang, X *et al*. [53]	2024	4	2	3	9
Coerkamp, C *et al*. [35]	2024	4	2	3	9

**Table 3.** **Quality evaluation form for case control studies**.

Study	Study	Selectiveness	Comparability	Exposure	Total score
Huang, M *et al*. [37]	2023	4	2	3	9
Sayama, K *et al*. [46]	2023	4	2	3	9
Li, D *et al*. (1) [39]	2025	4	2	3	9
Matsuda, K *et al*. [43]	2021	4	2	3	9

**Table 4.** **Quality evaluation form for cross-sectional study**.

Study	Study	Selectiveness	Comparability	Exposure	Total score
Xu, Q *et al*. [50]	2024	4	2	3	9

### 3.3 Meta-Analysis 

A total of 18 studies [33, 34, 35, 36, 37, 39, 40, 41, 42, 43, 44, 45, 46, 47, 48, 51, 52, 53] focused on the relationship between total FAI and MACE. Depending on the statistical methods used for FAI, FAI can be divided into two categories: the categorical variable and the continuous variable. In 12 studies [35, 39, 40, 41, 43, 44, 45, 46, 48, 51, 52, 53], FAI was used as a categorical variable. In 7 studies [33, 34, 36, 37, 42, 46, 47], FAI was used as a continuous variable. In one study [46], FAI was considered both a categorical variable and a continuous variable.

### 3.4 Correlation Between Total FAI and MACE (FAI as a Categorical Variable)

**Fig. 2.**
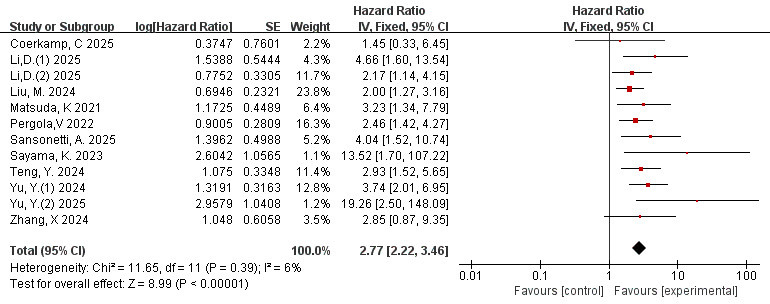
**Forest plots of the relationship between total FAI and MACE (FAI as a categorical variable)**. FAI, fat attenuation index; MACE, major adverse cardiovascular events.

A total of 12 studies [35, 39, 40, 41, 43, 44, 45, 46, 48, 51, 52, 53] evaluated the correlation between FAI and MACE as a categorical variable. The outcome of the Meta-analysis demonstrated a low extent of heterogeneity (I^2^ = 6%, *p* = 0.39). Therefore, the fixed effects model was employed. When FAI was used as a categorical variable, the risk of MACE in the high FAI group is 2.77 times higher than that in the low FAI group (HR = 2.77, 95% Cl = 2.22–3.46, *p* < 0.00001), and the difference was statistically significant (Fig. 2).

### 3.5 The Publication Bias of the Correlation Between Total FAI and MACE

**Fig. 3.**
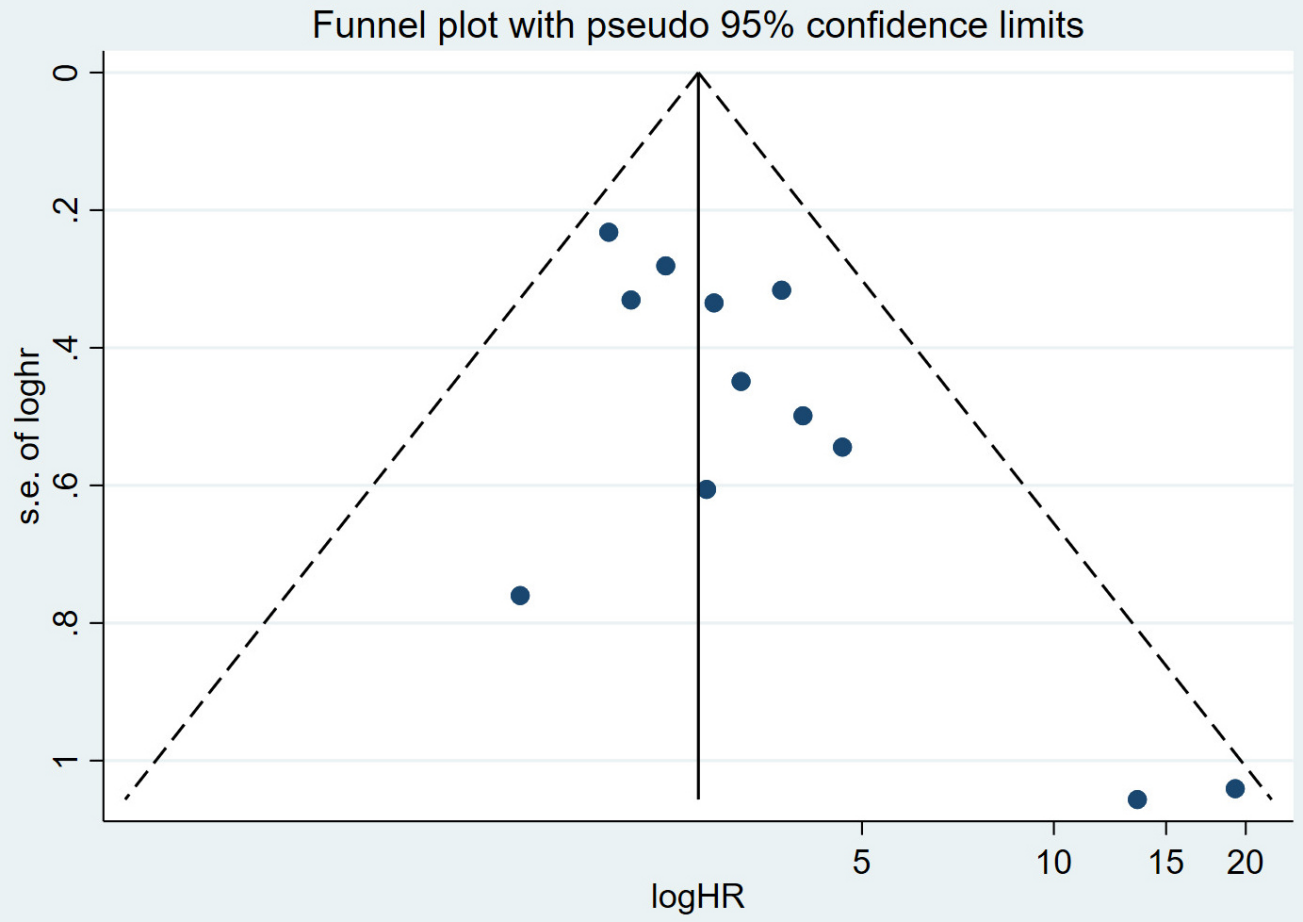
**Funnel plot of publication bias**.

The distribution of the scatter points in the funnel plot was not concentrated, and each scatter point was not symmetrical along the two sides of the dotted line, indicating that the publication bias is significant and the results may not be trustworthy (Fig. 3). We then performed Egger’s test, and *p* = 0.017. Due to *p* < 0.05, it suggests the presence of publication bias. Then, we used a trim-and-fill technique to further analyze the data. The number of missing studies estimated by the pruning method is 3. The combined result of the pre-pruning effect indicators is ln (HR) = 1.018, 95% CI = 0.80–1.24, and the combined result of the post-pruning effect indicators is ln (HR) = 2.586, 95% CI = 2.09–3.21, indicating a publication bias in the study. The original effect size may be underestimated, suggesting that the MACE risk is higher in the high FAI group than in the low FAI group. The reason for publication bias may be related to the small sample effect: (1) A small sample size can easily lead to random errors; (2) Small sample studies may yield negative results, and studies with smaller sample sizes are less likely to be accepted by journals. We removed individual studies one by one through sensitivity analysis, and the results remained stable, indicating that the results are relatively reliable.

### 3.6 Correlation Between Total FAI and MACE (FAI as a Continuous Variable) 

**Fig. 4.**
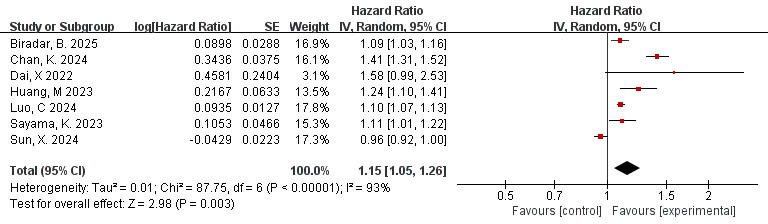
**Forest plots of the relationship between total FAI and MACE (FAI as a continuous variable)**.

7 studies [33, 34, 36, 37, 42, 46, 47] evaluated the correlation between FAI and MACE as a continuous variable. The result of the Meta-analysis showed a high level of heterogeneity (I^2^ = 93%, *p* < 0.00001). We conducted sensitivity analysis, but did not find significant clinical heterogeneity. Therefore, the random effects model was employed. When FAI is used as a continuous variable, the risk of MACE increases by 15% for each additional HU unit (HR = 1.15, 95% CI = 1.05–1.26, *p* = 0.003), and the difference was statistically significant (Fig. 4).

The reasons for heterogeneity may be as follows: (a) The statistical method of 1 study [36] is different from other studies, and its HR in this study is the effect of increasing the proportion of MACE per 10 HU increase, while in other studies it is the effect of increasing the proportion of MACE per 1 HU increase; (b) The different studies come from different research centers, with different instruments, measurement methods and statistical methods, which may cause deviations in the calculated results. The results of this meta-analysis are based on the heterogeneity caused by the experimental results, which have statistical differences and indicate a strong correlation between total FAI and MACE.

### 3.7 Correlation Between FAI of the RCA Branch and MACE (FAI as a Categorical Variable)

**Fig. 5.**
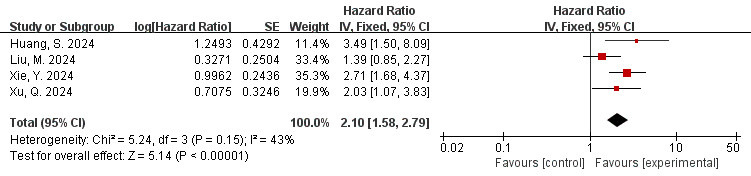
**Forest plots of the relationship between FAI of the RCA branch and MACE (FAI as a categorical variable)**. RCA, right coronary artery.

The data is derived from 4 studies [38, 41, 49, 50]. The outcome of the meta-analysis demonstrated a low extent of heterogeneity (I^2^ = 43%, *p* = 0.15). Thus, the fixed effects model was employed. As shown in Fig. 5, a significant association was observed between FAI measured in the RCA branch and adverse cardiovascular events (HR = 2.10, 95% CI = 1.58–2.79, *p* < 0.00001), and the difference was statistically significant (Fig. 5).

### 3.8 Correlation Between FAI of the RCA Branch and MACE (FAI as a Continuous Variable)

**Fig. 6.**
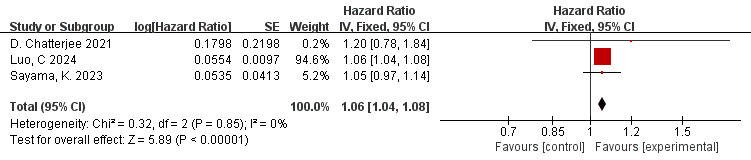
**Forest plots of the relationship between FAI of the RCA branch and MACE (FAI as a continuous variable)**.

The data is derived from 3 [10, 42, 46] studies. The findings of the meta-analysis revealed a low degree of heterogeneity (I^2^ = 0%, *p* = 0.85). Therefore, the fixed effects model was employed. The overall effect is shown in the figure: HR = 1.06, 95% CI = 1.04–1.08, *p* < 0.00001. A significant association was observed between FAI measured in the RCA and adverse cardiovascular events (Fig. 6).

### 3.9 Correlation Between FAI of the LAD Branch and MACE (FAI as a Categorical Variable)

**Fig. 7.**
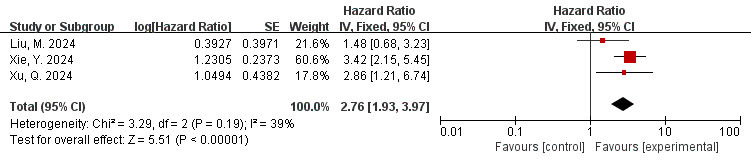
**Forest plots of the relationship between FAI of the LAD branch and MACE (FAI as a categorical variable)**. LAD, Left Anterior Descending artery.

The data is derived from 3 [41, 49, 50] studies. The findings of the meta-analysis revealed a low degree of heterogeneity (I^2^ = 39%, *p* = 0.19). Therefore, the fixed effects model was applied, indicating a low level of heterogeneity. The overall effect is shown in the figure: HR = 2.76, 95% CI = 1.93–3.97, *p* < 0.00001. A significant association was observed between FAI measured in the LAD and adverse cardiovascular events (Fig. 7).

### 3.10 Correlation Between FAI of the LAD Branch and MACE (FAI as a Continuous Variable)

**Fig. 8.**
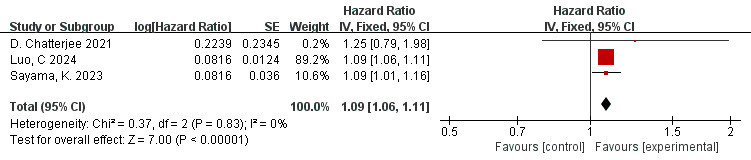
**Forest plots of the relationship between FAI of the LAD branch and MACE (FAI as a continuous variable)**.

The data is derived from 3 [10, 42, 46] studies. The meta-analysis indicated low heterogeneity (I^2^ = 0%, *p* = 0.83). Therefore, the fixed effects model was employed. The overall effect amount is shown in the figure: HR = 1.09, 95% CI = 1.06–1.11, *p* < 0.00001. There is a significant correlation between FAI of the LAD branch and adverse cardiovascular events (Fig. 8).

### 3.11 Correlation Between FAI of the LCX Branch and MACE (FAI as a Categorical Variable)

**Fig. 9.**
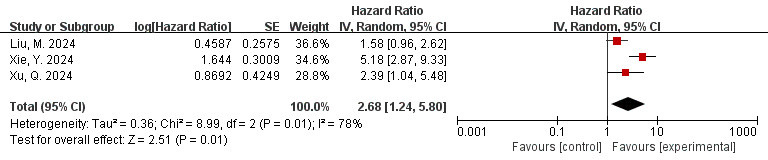
**Forest plots of the relationship between FAI of the LCX branch and MACE (FAI as a categorical variable)**. LCX, left circumflex coronary artery.

The data is derived from 3 [41, 49, 50] studies. The meta-analysis results indicated high heterogeneity (I^2^ = 78%, *p* = 0.01). Therefore, the random effects model was utilized. The overall effect is shown in the figure: HR = 2.68, 95% CI = 1.24–5.80, *p* = 0.01. A significant association was observed between FAI measured in the LCX and adverse cardiovascular events (Fig. 9).

### 3.12 Correlation Between FAI of the LCX Branch and MACE (FAI as a Continuous Variable)

**Fig. 10.**
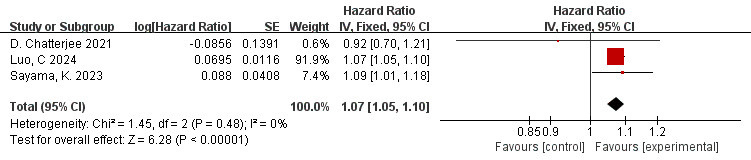
**Forest plots of the relationship between FAI of the LCX branch and MACE (FAI as a continuous variable)**.

The data is derived from 3 [10, 42, 46] studies. The findings of the meta-analysis revealed a low level of heterogeneity (I^2^ = 0%, *p* = 0.48). Therefore, the fixed effects model was applied. The overall amount is shown in the figure: HR = 1.07, 95% CI = 1.05–1.10, *p* < 0.00001. There is a significant correlation between FAI of the LCX branch and adverse cardiovascular events (Fig. 10).

## 4. Discussion

The objective of this meta-analysis is to explore the association between FAI and MACE. Our findings indicate a significant correlation between total FAI and branch FAI with MACE. Patients who have elevated FAI levels have a significantly higher risk of experiencing MACE. This result is consistent with findings from previous studies [54], further underscoring the importance of FAI in evaluating the risk of MACE.

FAI is a key imaging biomarker for quantifying the inflammatory status of peri-coronary adipose tissue and plaque lipid load, and plays an important role in the risk assessment of MACE [34]. Lesions with high FAI absolute values exhibit more frequent qualitative vulnerable plaque features than those with lower FAI absolute values. The rupture of vulnerable plaques results in thrombosis, directly leading to acute MACE events [55]. Clinical studies have found that in patients with coronary heart disease, the FAI is significantly correlated with the degree of coronary stenosis and closely related to plaque stability. Since it is a quantitative evaluation indicator, it can provide reliable clinical evidence for early screening of high-risk plaques and effectively predict the risk of MACE, providing support for clinical risk stratification and determining the need for interventional procedures [56, 57]. Therefore, coronary artery CT angiography FAI may be a potentially important predictor for MACE. Assessing the relationship between FAI and MACE has significant clinical importance.

There have been several similar meta-analyses on this subject in the past [32, 57, 58]. However, the previous meta-analysis included relatively little literature and did not consider several recent important findings. Compared with the previous meta-analysis, we believe that the results of the current meta-analysis exploring the association of FAI with MACE are the most recent with more included studies and a larger sample size. In addition, we also reviewed different types of FAI. Therefore, our conclusion appears to be more comprehensive.

However, this study also has some limitations: (1) Despite a comprehensive literature review, it remains challenging to fully rule out the risk of publication bias, as studies with certain negative findings may be excluded due to being unpublished, which could potentially compromise the accuracy of the results. (2) Measurement methods and definitions for FAI are not fully standardized, and there is some heterogeneity in the definition of adverse cardiovascular events, which might impact the precision of the outcomes. (3) Given that most research adopts an observational design, it is difficult to ascertain the causal relationship between FAI and adverse cardiovascular events. In addition, high-quality prospective studies are necessary to further confirm the observed association. (4) The subgroup analysis of each coronary artery branch (RCA, LAD, LCX) is based on only 3 or 4 studies, with relatively limited sample sizes. The results are only preliminary exploratory findings, and their external validity may be limited. Additional high-quality research is required to further verify the findings. (5) The research results of FAI as a categorical variable belong to exploratory analysis, whose purpose is not to obtain an exact combined effect quantity, but to preliminarily explore whether there is a rough directional correlation between abnormal FAI levels (regardless of the cut-off point definition) and MACE.

The results of this study have important clinical implications, suggesting that clinicians should consider FAI as an indicator when assessing the risk of cardiovascular disease. Incorporating FAI into the cardiovascular disease risk assessment system may help to better identify high-risk populations and develop more targeted prevention and treatment strategies. For individuals with elevated FAI levels, lifestyle interventions such as a balanced diet and increased physical activity can be used to reduce FAI levels and thereby reduce the risk of cardiovascular disease. Additional large-scale, multicenter, prospective studies are required to further elucidate the causal association and underlying mechanisms between FAI and MACE. Further efforts should be made to develop more accurate and convenient methods for FAI detection to promote their widespread use in clinical practice.

## 5. Conclusion

Our meta-analysis provides preliminary evidence supporting a significant correlation between FAI and MACE, with individuals with elevated FAI levels having a significantly increased risk of developing MACE. However, due to potential biases in the included studies, the evidence may be limited and further evaluation is needed.

## Data Availability

All relevant data generated or analyzed as part of this study are included in the article and the attachments. The original data of the studies included in this research can be retrieved from their respective published articles and supplementary materials.

## Abbreviations

ACS, acute coronary syndrome; CAD, coronary artery disease; CCS, chronic coronary syndromes; CCTA, CT angiography; CI, confidence interval; CVDs, cardiovascular diseases; FAI, fat attenuation index; HU, Hounsfield unit; HR, hazard ratios; LAD, left anterior descending; LCX, left circumflex coronary artery; MACE, major adverse cardiovascular events; MACCE, major adverse cardiac and cerebrovascular events; non-IR UMI, non-infarct-related territory unrecognized myocardial infarction; NOS, Newcastle-Ottawa scale; PVAT, perivascular adipose tissue; RCA, right coronary artery; RCTs, randomized controlled trials.

## Supplementary Material

Supplementary material associated with this article can be found, in the online version, at https://doi.org/10.31083/RCM46683.
