# Supplementary material for: Correlation Between Fat Attenuation Index and Major Adverse Cardiovascular Events: A Systematic Review and Meta-Analysis
Source: Rev Cardiovasc Med. 2026 Apr 24;27(4):46683. doi: 10.31083/RCM46683 (PMC13155992; doi:10.31083/RCM46683)
Supplement: Supplementary file 1 [file 2153-8174-27-4-46683-s1.zip › Supplement Material 2.docx]

**search strategy**

**PubMed**

| Search | Query | number of documents |
| --- | --- | --- |
| #1 | Search: **(Fat Attenuation Index) OR (FAI)** | 7143 |
| #2 | Search:(Cardiovascular Diseases[Mesh]) OR (adverse cardiac events) OR (cardiovascular events) OR (Cardiovascular Disease) OR (Disease, Cardiovascular) OR (Cardiac Events) OR (Cardiac Event) OR (Event, Cardiac) OR (Adverse Cardiac Event) OR (Cardiovascular Diseases) OR (Cardiac Event, Adverse) OR (Cardiac Events, Adverse) OR (Major Adverse Cardiac Events) OR (MACE) | 3210779 |
| #3 | Search: (Myocardial Infarction[Mesh]) OR (Infarction, Myocardial) OR (Infarctions, Myocardial) OR (Myocardial Infarctions) OR (Heart Attack) OR (Heart Attacks) OR (Myocardial Infarct) OR (Infarct, Myocardial) OR (Infarcts, Myocardial) OR (Myocardial Infarcts) OR (Cardiovascular Stroke) OR (Cardiovascular Strokes) OR (Stroke, Cardiovascular) OR (Strokes, Cardiovascular) OR(Myocardial Infarction) OR (Shock, Cardiogenic) OR (Cardiogenic Shock) | 408569 |
| #4 | Search: (Stroke[Mesh]) OR (Strokes) OR (Cerebrovascular Accident) OR (Cerebrovascular Accidents) OR (Cerebral Stroke) OR (Cerebral Strokes) OR (Stroke, Cerebral) OR (Strokes, Cerebral) OR (Cerebrovascular Apoplexy) OR (Apoplexy, Cerebrovascular) OR (Vascular Accident, Brain) OR (Brain Vascular Accident) OR (Brain Vascular Accidents) OR (Vascular Accidents, Brain) OR (Cerebrovascular Stroke) OR (Cerebrovascular Strokes) OR (Stroke, Cerebrovascular) OR (Strokes, Cerebrovascular) OR (Apoplexy) OR (CVA) OR (Cerebrovascular Accident) OR (CVAs) OR (Cerebrovascular Accident) OR (Stroke, Acute) OR (Acute Stroke) OR (Acute Strokes) OR (Strokes, Acute) OR (Cerebrovascular Accident, Acute) OR (Acute Cerebrovascular Accident) OR (Acute Cerebrovascular Accidents) OR (Cerebrovascular Accidents, Acute) | 511959 |
| #5 | Search: (Heart Failure[Mesh]) OR (Cardiac Failure) OR (Heart Decompensation) OR (Decompensation, Heart) OR (Congestive Heart Failure) OR (Heart Failure, Congestive) OR (Heart Failure, Right-Sided) OR (Heart Failure, Right Sided) OR (Right-Sided Heart Failure) OR (Right Sided Heart Failure) OR (Heart Failure, Left-Sided) OR (Heart Failure, Left Sided) OR (Left-Sided Heart Failure) OR (Left Sided Heart Failure) OR (Myocardial Failure) OR (Heart Failure) | 390563 |
| #6 | Search: (Death, Sudden, Cardiac[Mesh]) OR (Cardiac Sudden Death) OR (Death, Cardiac Sudden) OR (Sudden Death, Cardiac) OR (Sudden Cardiac Death) OR (Cardiac Death, Sudden) OR (Death, Sudden Cardiac) OR (Sudden Cardiac Arrest) OR (Arrest, Sudden Cardiac) OR (Cardiac Arrests, Sudden) OR (Cardiac Arrest, Sudden) | 45984 |
| #7 | Search: (Heart Arrest[Mesh]) OR (Heart arrest) OR（Arrest, Heart）OR (Asystole) OR (Asystoles) OR (Cardiac Arrest) OR (Arrest,Cardiac)OR (Cardiopulmonary Arrest) OR (Arrest, Cardiopulmonary) | 102038 |
| #8 | Search: (Myocardial Revascularization[Mesh]) OR (Myocardial Revascularizations) OR (Revascularization) OR (Revascularization, Myocardial) OR (Revascularizations, Myocardial) OR (urgent Revascularization) OR (Internal Mammary Artery Implantation) OR (Myocardial Revascularization) OR (coronary angioplasty) OR (directional coronary atherectomy) OR (coronary artery bypass) | 186529 |
| #9 | Search: (Arrhythmias, Cardiac[Mesh]) OR (Severe arrhythmia) OR (Arrhythmia, Cardiac) OR (Arrhythmia) OR (Arrythmia) OR (Cardiac Arrhythmia) OR (Cardiac Arrhythmias) OR (Cardiac Dysrhythmia) OR (Dysrhythmia, Cardiac) OR (Arrhythmias, cardiac) OR (Arrhythmia, Sinus[Mesh]) OR (Arrhythmia, Sinus) OR (Atrial Fibrillation[Mesh]) OR (Atrial Fibrillation) OR (Atrial Flutter[Mesh]) OR (Atrial Flutter) | 351552 |
| #10 | Search: (Angina Pectoris[Mesh]) OR (Angor Pectoris) OR (Stenocardia) OR (Stenocardias) OR (Recurrent angina pectoris) OR (Angina Pectoris) OR (Angina Pectoris) OR (Angina) | 78008 |
| #11 | Search: #2 OR #3 OR #4 OR #5 OR #6 OR #7 OR #8 OR #9 OR #10 | 3562284 |
| #12 | Search: #1 AND #11 | 982 |

**EMbase**

| Search | Query | number of documents |
| --- | --- | --- |
| #1 | Search: **(****Fat Attenuation Index) OR (FAI)** | 20738 |
| #2 | Search:‘cardiovascular disease’[Mesh] OR 'angiocardiopathy' OR 'angiocardiovascular disease' OR 'cardiovascular complication' OR 'cardiovascular diseases' OR 'cardiovascular disorder' OR 'cardiovascular disturbance' OR 'cardiovascular lesion' OR 'cardiovascular syndrome' OR 'cardiovascular vegetative disorder' OR 'complication, cardiovascular' OR 'disease, cardiovascular' OR 'major adverse cardiovascular event' OR 'cardiovascular disease' OR ‘MACE’ | 6271231 |
| #3 | Search: ‘heart infarction’[Mesh] OR 'cardiac infarct' OR 'cardiac infarction' OR 'cardial infarct' OR 'heart attack' OR 'heart infarct' OR 'heart micro infarction' OR 'heart muscle infarction' OR 'infarction, heart' OR 'myocardial infarct' OR 'myocardial infarction' OR 'myocardium infarct' OR 'myocardium infarction' OR 'premonitory infarction sign' OR 'second heart attack' OR 'subendocardial infarction' OR 'transmural cardiac infarction' OR 'transmural heart infarction' OR 'transmural infarction, heart' OR 'heart infarction' | 582306 |
| #4 | Search: ‘cerebrovascular accident’[Mesh] OR 'accident, cerebrovascular' OR 'acute cerebrovascular lesion' OR 'acute focal cerebral vasculopathy' OR 'acute stroke' OR 'apoplectic stroke' OR 'apoplexia' OR 'apoplexy' OR 'blood flow disturbance, brain' OR 'brain accident' OR 'brain attack' OR 'brain blood flow disturbance' OR 'brain insult' OR 'brain insultus' OR 'brain vascular accident' OR 'cerebral apoplexia' OR 'cerebral insult' OR 'cerebral stroke' OR 'cerebral vascular accident' OR 'cerebral vascular insufficiency' OR 'cerebro vascular accident' OR 'cerebrovascular arrest' OR 'cerebrovascular failure' OR 'cerebrovascular injury' OR 'cerebrovascular insufficiency' OR 'cerebrovascular insult' OR 'cerebrum vascular accident' OR 'cryptogenic stroke' OR 'CVA' OR 'insultus cerebralis' OR 'ischaemic seizure' OR 'ischemic seizure' OR 'stroke' OR 'thrombotic stroke' OR 'cerebrovascular accident' | 810591 |
| #5 | Search: (Heart Failure[Mesh]) OR 'backward failure, heart' OR 'cardiac backward failure' OR 'cardiac decompensation' OR 'cardiac failure' OR 'cardiac incompetence' OR 'cardiac insufficiency' OR 'cardiac stand still' OR 'cardial decompensation' OR 'cardial insufficiency' OR 'chronic heart failure' OR 'chronic heart insufficiency' OR 'decompensatio cordis' OR 'decompensation, heart' OR 'heart backward failure' OR 'heart decompensation' OR 'heart incompetence' OR 'heart insufficiency' OR 'insufficientia cardis' OR 'myocardial failure' OR 'myocardial insufficiency' OR 'heart failure' | 869042 |
| #6 | Search: ‘sudden cardiac death’[Mesh]) OR 'cardiac death, sudden' OR 'cardiac sudden death' OR 'death, sudden, cardiac' OR 'sudden cardiac arrest' OR 'sudden heart death' OR 'sudden cardiac death' | 52627 |
| #7 | Search: ‘Heart Arrest’[Mesh]) OR 'arrest, heart' OR 'asystole' OR 'asystolia' OR 'asystoly' OR 'cardiac arrest' OR 'circulation arrest' OR 'circulatory arrest' OR 'heart arrest, induced' OR 'heart asystole' OR 'heart standstill' OR 'induced heart arrest' OR 'heart arrest' | 174572 |
| #8 | Search: ‘heart muscle revascularization’[Mesh] OR 'anastomosis, internal mammary artery' OR 'artery implantation, mammary' OR 'cardiac muscle revascularisation' OR 'cardiac muscle revascularization' OR 'coronary revascularisation' OR 'coronary revascularization' OR 'heart muscle revascularisation' OR 'heart myocardium revascularisation' OR 'heart revascularisation' OR 'heart revascularization' OR 'implantation, internal mammary artery' OR 'internal mammary arterial anastomosis' OR 'internal mammary arterial implantation' OR 'internal mammary artery anastomosis' OR 'internal mammary artery graft' OR 'internal mammary artery implant' OR 'internal mammary artery implantation' OR 'internal mammary artery reimplantation' OR 'internal mammary-coronary artery anastomosis' OR 'mammary arterial implantation' OR 'mammary artery implantation' OR 'myocardial revascularisation' OR 'myocardial revascularization' OR 'myocardium revascularisation' OR 'myocardium revascularization' OR 'revascularisation, transmyocardial laser' OR 'revascularization, transmyocardial laser' OR 'transmyocardial laser revascularisation' OR 'transmyocardial laser revascularization' OR 'vineberg operation' OR 'heart muscle revascularization' | 50908 |
| #9 | Search: ‘heart arrhythmia’[Mesh] OR ‘sinus arrhythmia’ [Mesh] OR ‘Atrial Fibrillation’[Mesh] OR ‘heart atrium flutter’[Mesh] OR 'arrhythmia' OR 'arrhythmias, cardiac' OR 'arrhytmia, heart' OR 'cardiac arrhythmia' OR 'cardiac arrhythmias' OR 'cardiac arrythmia' OR 'cardiac disrhythmia' OR 'cardiac dysrhythmia' OR 'cardial arrhythmia' OR 'ectopic heart rhythm' OR 'ectopic rhythm' OR 'heart aberrant conduction' OR 'heart arrhytmia' OR 'heart arrythmia' OR 'heart dysrhythmia' OR 'heart ectopic beat' OR 'heart ectopic ventricle contraction' OR 'heart rhythm disease' OR 'heart rhythm disorder' OR 'heart rhythm problem' OR 'myocardial arrhythmia' OR 'heart arrhythmia' OR 'arrhythmia, sinus' OR 'SA nodal arrhythmia' OR 'SA node arrhythmia' OR 'sino-atrial node arrhythmia' OR 'sinoatrial node arrhythmia' OR 'sinus arrhythmica' OR 'sinus node arrhythmia' OR 'sinus node syndrome' OR 'sinus arrhythmia'OR 'atrium fibrillation' OR 'auricular fibrilation' OR 'auricular fibrillation' OR 'cardiac atrial fibrillation' OR 'cardiac atrium fibrillation' OR 'fibrillation, heart atrium' OR 'heart atrial fibrillation' OR 'heart atrium fibrillation' OR 'heart fibrillation atrium' OR 'non-valvular atrial fibrillation' OR 'nonvalvular atrial fibrillation' OR 'atrial fibrillation' OR 'atrial flutter' OR 'atrium flutter' OR 'atrium flutter, heart' OR 'auricular flutter' OR 'cardiac atrial flutter' OR 'cardiac atrium flutter' OR 'flutter, heart atrium' OR 'heart atrial flutter' OR 'supraventricular flutter' OR 'heart atrium flutter' | 826849 |
| #10 | Search: ‘Angina Pectoris’[Mesh] OR 'angina' OR 'anginal attack' OR 'stenocardia' OR 'angina pectoris' | 148746 |
| #11 | Search: #2 OR #3 OR #4 OR #5 OR #6 OR #7 OR #8 OR #9 OR #10 | 6429786 |
| #12 | Search: #1 AND #11 | 3364 |

**Web of Science**

| Search | Query | number of documents |
| --- | --- | --- |
| #1 | Search: **(Fat Attenuation Index) OR (FAI)** | 7692 |
| #2 | Search: (adverse cardiac events) OR (cardiovascular events) OR (Cardiovascular Disease) OR (Disease, Cardiovascular) OR (Cardiac Events) OR (Cardiac Event) OR (Event, Cardiac) OR (Adverse Cardiac Event) OR (Cardiovascular Diseases) OR (Cardiac Event, Adverse) OR (Cardiac Events, Adverse) OR (Major Adverse Cardiac Events) OR (MACE) | 2845073 |
| #3 | Search: (Infarction, Myocardial) OR (Infarctions, Myocardial) OR (Myocardial Infarctions) OR (Heart Attack) OR (Heart Attacks) OR (Myocardial Infarct) OR (Infarct, Myocardial) OR (Infarcts, Myocardial) OR (Myocardial Infarcts) OR (Cardiovascular Stroke) OR (Cardiovascular Strokes) OR (Stroke, Cardiovascular) OR (Strokes, Cardiovascular) OR(Myocardial Infarction) OR (Shock, Cardiogenic) OR (Cardiogenic Shock) | 825556 |
| #4 | Search: (Strokes) OR (Cerebrovascular Accident) OR (Cerebrovascular Accidents) OR (Cerebral Stroke) OR (Cerebral Strokes) OR (Stroke, Cerebral) OR (Strokes, Cerebral) OR (Cerebrovascular Apoplexy) OR (Apoplexy, Cerebrovascular) OR (Vascular Accident, Brain) OR (Brain Vascular Accident) OR (Brain Vascular Accidents) OR (Vascular Accidents, Brain) OR (Cerebrovascular Stroke) OR (Cerebrovascular Strokes) OR (Stroke, Cerebrovascular) OR (Strokes, Cerebrovascular) OR (Apoplexy) OR (CVA) OR (Cerebrovascular Accident) OR (CVAs) OR (Cerebrovascular Accident) OR (Stroke, Acute) OR (Acute Stroke) OR (Acute Strokes) OR (Strokes, Acute) OR (Cerebrovascular Accident, Acute) OR (Acute Cerebrovascular Accident) OR (Acute Cerebrovascular Accidents) OR (Cerebrovascular Accidents, Acute) | 746828 |
| #5 | Search: (Heart Failure[Mesh]) OR (Cardiac Failure) OR (Heart Decompensation) OR (Decompensation, Heart) OR (Congestive Heart Failure) OR (Heart Failure, Congestive) OR (Heart Failure, Right-Sided) OR (Heart Failure, Right Sided) OR (Right-Sided Heart Failure) OR (Right Sided Heart Failure) OR (Heart Failure, Left-Sided) OR (Heart Failure, Left Sided) OR (Left-Sided Heart Failure) OR (Left Sided Heart Failure) OR (Myocardial Failure) OR (Heart Failure) | 678289 |
| #6 | Search: (Cardiac Sudden Death) OR (Death, Cardiac Sudden) OR (Sudden Death, Cardiac) OR (Sudden Cardiac Death) OR (Cardiac Death, Sudden) OR (Death, Sudden Cardiac) OR (Sudden Cardiac Arrest) OR (Arrest, Sudden Cardiac) OR (Cardiac Arrests, Sudden) OR (Cardiac Arrest, Sudden) | 73187 |
| #7 | Search: (Heart arrest) OR（Arrest, Heart）OR (Asystole) OR (Asystoles) OR (Cardiac Arrest) OR (Arrest,Cardiac)OR (Cardiopulmonary Arrest) OR (Arrest, Cardiopulmonary) | 127771 |
| #8 | Search: (Myocardial Revascularizations) OR (Revascularization) OR (Revascularization, Myocardial) OR (Revascularizations, Myocardial) OR (urgent Revascularization) OR (Internal Mammary Artery Implantation) OR (Myocardial Revascularization) OR (coronary angioplasty) OR (directional coronary atherectomy) OR (coronary artery bypass) | 247634 |
| #9 | Search: (Severe arrhythmia) OR (Arrhythmia, Cardiac) OR (Arrhythmia) OR (Arrythmia) OR (Cardiac Arrhythmia) OR (Cardiac Arrhythmias) OR (Cardiac Dysrhythmia) OR (Dysrhythmia, Cardiac) OR (Arrhythmias, cardiac) OR (Arrhythmia, Sinus) OR (Atrial Fibrillation) OR (Atrial Flutter) | 390541 |
| #10 | Search: (Angor Pectoris) OR (Stenocardia) OR (Stenocardias) OR (Recurrent angina pectoris) OR (Angina Pectoris) OR (Angina Pectoris) OR (Angina) | 110165 |
| #11 | Search: #2 OR #3 OR #4 OR #5 OR #6 OR #7 OR #8 OR #9 OR #10 | 4091369 |
| #12 | Search: #1 AND #11 | 1006 |

**The Cochrane library**

| Search | Query | number of documents |
| --- | --- | --- |
| #1 | Search: **(Fat Attenuation Index) OR (FAI)** | 875 |
| #2 | Search:(Cardiovascular Diseases[Mesh]) OR (adverse cardiac events) OR (cardiovascular events) OR (Cardiovascular Disease) OR (Disease, Cardiovascular) OR (Cardiac Events) OR (Cardiac Event) OR (Event, Cardiac) OR (Adverse Cardiac Event) OR (Cardiovascular Diseases) OR (Cardiac Event, Adverse) OR (Cardiac Events, Adverse) OR (Major Adverse Cardiac Events) OR (MACE) | 219148 |
| #3 | Search: (Myocardial Infarction[Mesh]) OR (Infarction, Myocardial) OR (Infarctions, Myocardial) OR (Myocardial Infarctions) OR (Heart Attack) OR (Heart Attacks) OR (Myocardial Infarct) OR (Infarct, Myocardial) OR (Infarcts, Myocardial) OR (Myocardial Infarcts) OR (Cardiovascular Stroke) OR (Cardiovascular Strokes) OR (Stroke, Cardiovascular) OR (Strokes, Cardiovascular) OR(Myocardial Infarction) OR (Shock, Cardiogenic) OR (Cardiogenic Shock) | 53142 |
| #4 | Search: (Stroke[Mesh]) OR (Strokes) OR (Cerebrovascular Accident) OR (Cerebrovascular Accidents) OR (Cerebral Stroke) OR (Cerebral Strokes) OR (Stroke, Cerebral) OR (Strokes, Cerebral) OR (Cerebrovascular Apoplexy) OR (Apoplexy, Cerebrovascular) OR (Vascular Accident, Brain) OR (Brain Vascular Accident) OR (Brain Vascular Accidents) OR (Vascular Accidents, Brain) OR (Cerebrovascular Stroke) OR (Cerebrovascular Strokes) OR (Stroke, Cerebrovascular) OR (Strokes, Cerebrovascular) OR (Apoplexy) OR (CVA) OR (Cerebrovascular Accident) OR (CVAs) OR (Cerebrovascular Accident) OR (Stroke, Acute) OR (Acute Stroke) OR (Acute Strokes) OR (Strokes, Acute) OR (Cerebrovascular Accident, Acute) OR (Acute Cerebrovascular Accident) OR (Acute Cerebrovascular Accidents) OR (Cerebrovascular Accidents, Acute) | 58093 |
| #5 | Search: (Heart Failure[Mesh]) OR (Cardiac Failure) OR (Heart Decompensation) OR (Decompensation, Heart) OR (Congestive Heart Failure) OR (Heart Failure, Congestive) OR (Heart Failure, Right-Sided) OR (Heart Failure, Right Sided) OR (Right-Sided Heart Failure) OR (Right Sided Heart Failure) OR (Heart Failure, Left-Sided) OR (Heart Failure, Left Sided) OR (Left-Sided Heart Failure) OR (Left Sided Heart Failure) OR (Myocardial Failure) OR (Heart Failure) | 55852 |
| #6 | Search: (Death, Sudden, Cardiac[Mesh]) OR (Cardiac Sudden Death) OR (Death, Cardiac Sudden) OR (Sudden Death, Cardiac) OR (Sudden Cardiac Death) OR (Cardiac Death, Sudden) OR (Death, Sudden Cardiac) OR (Sudden Cardiac Arrest) OR (Arrest, Sudden Cardiac) OR (Cardiac Arrests, Sudden) OR (Cardiac Arrest, Sudden) | 3036 |
| #7 | Search: (Heart Arrest[Mesh]) OR (Heart arrest) OR（Arrest, Heart）OR (Asystole) OR (Asystoles) OR (Cardiac Arrest) OR (Arrest,Cardiac)OR (Cardiopulmonary Arrest) OR (Arrest, Cardiopulmonary) | 7992 |
| #8 | Search: (Myocardial Revascularization[Mesh]) OR (Myocardial Revascularizations) OR (Revascularization) OR (Revascularization, Myocardial) OR (Revascularizations, Myocardial) OR (urgent Revascularization) OR (Internal Mammary Artery Implantation) OR (Myocardial Revascularization) OR (coronary angioplasty) OR (directional coronary atherectomy) OR (coronary artery bypass) | 31550 |
| #9 | Search: (Arrhythmias, Cardiac[Mesh]) OR (Severe arrhythmia) OR (Arrhythmia, Cardiac) OR (Arrhythmia) OR (Arrythmia) OR (Cardiac Arrhythmia) OR (Cardiac Arrhythmias) OR (Cardiac Dysrhythmia) OR (Dysrhythmia, Cardiac) OR (Arrhythmias, cardiac) OR (Arrhythmia, Sinus[Mesh]) OR (Arrhythmia, Sinus) OR (Atrial Fibrillation[Mesh]) OR (Atrial Fibrillation) OR (Atrial Flutter[Mesh]) OR (Atrial Flutter) | 31836 |
| #10 | Search: (Angina Pectoris[Mesh]) OR (Angor Pectoris) OR (Stenocardia) OR (Stenocardias) OR (Recurrent angina pectoris) OR (Angina Pectoris) OR (Angina Pectoris) OR (Angina) | 16416 |
| #11 | Search: #2 OR #3 OR #4 OR #5 OR #6 OR #7 OR #8 OR #9 OR #10 | 291989 |
| #12 | Search: #1 AND #11 | 173 |
